# Skyrmion Phase in MnSi Thin Films Grown on Sapphire by a Conventional Sputtering

**DOI:** 10.1186/s11671-020-03462-2

**Published:** 2021-01-06

**Authors:** Won-Young Choi, Hyun-Woo Bang, Seung-Hyun Chun, Sunghun Lee, Myung-Hwa Jung

**Affiliations:** 1 Department of Physics, Sogang University, Seoul, 04107 Korea; 2 Department of Physics, Sejong University, Seoul, 05006 Korea

**Keywords:** MnSi, Sputtering, Polycrystal, Skyrmion, Topological Hall effect

## Abstract

Topologically protected chiral skyrmions are an intriguing spin texture that has attracted much attention because of fundamental research and future spintronic applications. MnSi with a non-centrosymmetric structure is a well-known material hosting a skyrmion phase. To date, the preparation of MnSi crystals has been investigated by using special instruments with an ultrahigh vacuum chamber. Here, we introduce a facile way to grow MnSi films on a sapphire substrate using a relatively low vacuum environment of conventional magnetron sputtering. Although the as-grown MnSi films have a polycrystalline nature, a stable skyrmion phase in a broad range of temperatures and magnetic fields is observed via magnetotransport properties including phenomenological scaling analysis of the Hall resistivity contribution. Our findings provide not only a general way to prepare the materials possessing skyrmion phases but also insight into further research to stimulate more degrees of freedom in our inquisitiveness.

## Introduction

Topologically protected chiral skyrmions have a vortex-like nontrivial swirling spin texture, where magnetic spins stabilized by Dzyaloshinskii–Moriya interaction (DMI) align in a non-collinear manner surrounding a sphere [1]. A large DMI is generally induced in non-centrosymmetric ferromagnets, owing to the broken inversion symmetry [2]. This complex spin texture has garnered massive attention because of the intriguing physical properties for both fundamental research and possible applications in future technology [3, 4]. Compared to magnetic domain walls, skyrmion domains exhibit stable current-driven motion at remarkably low current density, enabling low-power consumption spintronic devices [5].

MnSi with a non-centrosymmetric B20 phase is an archetypal helimagnetic material hosting a skyrmionic lattice, which has been studied theoretically and experimentally for decades [6–10]. In the skyrmionic lattice of MnSi, spin transfer torque (STT) is observed, leading to further investigations on the injection of spin-polarized currents [5]. In particular, the skyrmion size of MnSi ranges from ~ 18 nm, which is considered small among well-known groups with skyrmion spin textures [11]. STT tends to increase significantly with reducing skyrmion size [12, 13]. Although material parameters affect the skyrmion size, DMI and ferromagnetic exchange interaction mainly contribute to determining the skyrmion size [14]. In this regard, MnSi has excellent prospects as a good candidate for applied physics.

To confirm the evident skyrmions, diverse measurement tools, such as Lorentz transmission electron microscopy, magnetic transmission soft X-ray microscopy, magnetic force microscopy, and small-angle neutron scattering, have been used [15–18]. Such microscopic tools allow direct identification of the skyrmionic lattice in real-space, but high-quality single crystals or epitaxial thin films are needed, which are grown by special instruments with a high-vacuum chamber. The other way to reveal the existence of skyrmions is to measure the magnetotransport properties and the topological Hall effect (THE), as shown in previous reports [9, 19–21]. Skyrmions can be observed even in polycrystalline samples because they are topological objects in which the topological phase is less susceptible to impurities or crystalline nature [22].

Here, we report the magnetotransport properties of polycrystalline MnSi grown by conventional sputtering. We employed X-ray diffraction (XRD) and transmission electron microscopy (TEM) to identify the single phase of MnSi crystals and their crystallinity. The magnetic transition at approximately 25 K was revealed by measuring temperature-dependent magnetization and resistance curves, where magnetoresistance data also exhibited a distinguishable shape at the border of the transition temperature. We successfully extracted the THE signal from the measured Hall resistance, and plotted contour mapping of topological Hall resistivity as a function of temperature and magnetic field. Moreover, the analysis of the anomalous Hall resistivity contribution in MnSi films implied the stabilization of the skyrmion phase in a broader range of temperatures and magnetic fields, albeit impurities and defects in the polycrystalline MnSi sample. Our findings show that the skyrmions can be observed in polycrystalline MnSi films grown by facile and inexpensive instruments, and further investigations of similar materials possessing skyrmionic lattices can be stimulated.

## Methods

MnSi films were deposited on Si (001) and *c*-cut sapphire (Al_2_O_3_) substrates by direct current (DC)/radio frequency (RF) magnetron sputtering with a base pressure of 1.0 × 10^–6^ Torr. The MnSi films were grown at room temperature under 10 mTorr Ar pressure by co-sputtering Mn and Si targets for 5 min. The DC power for the Mn target was 10 to 20 W, and the RF power for the Si target was 100 W. Following the deposition of MnSi, the as-grown MnSi was crystallized by inducing an in situ annealing treatment for 2 h in the temperature range of 550–590 °C. The crystal phase and structure of the samples were examined by XRD with an X-ray source of Mo and Ag at 60 kV. The morphological characterization and chemical composition of the samples were analyzed by scanning electron microscopy (SEM), atomic force microscopy (AFM), and high-resolution transmission electron microscopy (HR-TEM) equipped with energy-dispersive spectroscopy (EDS). The magnetic and electrical properties were measured using a superconducting quantum interference device-vibrating sample magnetometer (SQUID-VSM), where the magnetic field and temperature were swept up to 50 kOe and down to 2 K, respectively.

## Results and Discussion

The growth of MnSi films has been well described in previous reports with various methods [2, 9, 21–25]. However, most techniques to grow MnSi require specific facilities with an ultrahigh vacuum environment, while development of conventional magnetron sputtering with a relatively low base pressure has not yet been introduced. Since the lattice mismatch between the Si (001) substrate and cubic MnSi structure is estimated to be approximately 19%, we tested the optimal growth conditions of the MnSi films on Si (001) substrates. A co-sputtering method with Mn and Si targets was employed, and growth conditions such as RF power, growth temperature, and annealing treatments were minutely controlled to grow the MnSi films (Additional file 1: Table S1). Aguf et al*.* reported that as-deposited MnSi films were amorphous unless they were crystallized by annealing treatment [23]. Indeed, we found that the initially deposited amorphous MnSi turned into a crystallized MnSi phase after annealing treatment (Additional file 1: Fig. S1). Most results using Si (001) substrates, however, showed that mixed phases of MnSi and Mn_5_Si_3_ were observed by XRD measurements. For this reason, Si (001) substrates were replaced by Al_2_O_3_ substrates having a low lattice mismatch (~ 4.2%).

**Fig. 1:**
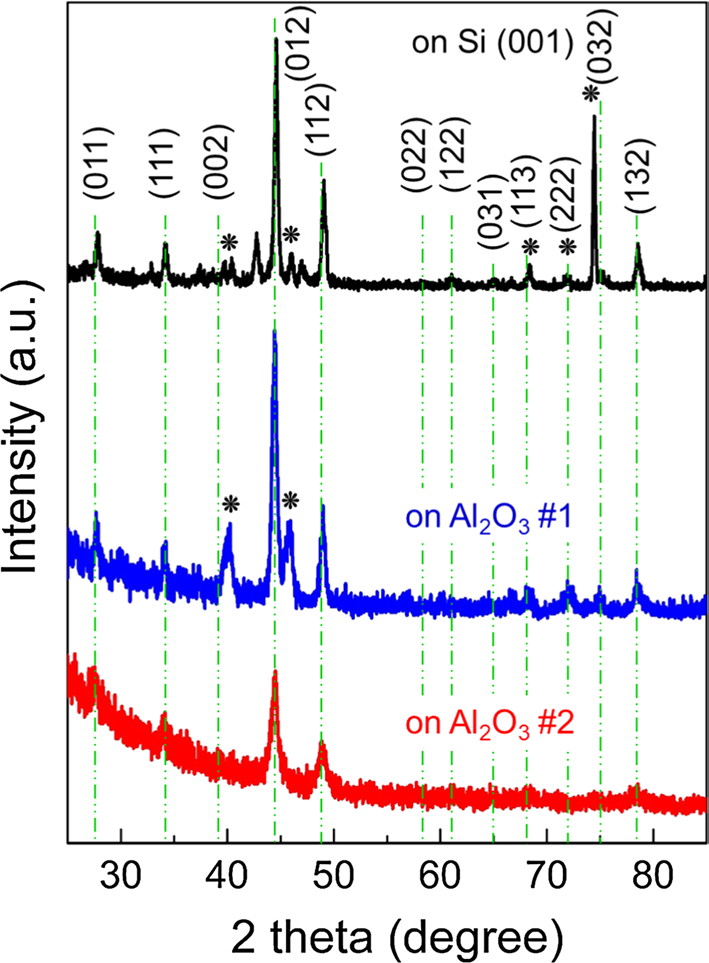
XRD patterns of MnSi films on Si [(001), black solid line] and Al_2_O_3_ (blue and red solid lines) substrates. All the peaks are indexed to the cubic B20-type MnSi phase, marked with green dotted lines. The asterisks on black and blue solid lines indicate peaks from the Mn_5_Si_3_ phase

Figure 1 presents the XRD patterns of the MnSi films grown on Si (black solid line) and Al_2_O_3_ (blue and red solid lines) substrates, where the MnSi films on Si (001) and on Al_2_O_3_ #1 were deposited under the same growth conditions (15 W power for Mn, 100 W power for Si, 590 °C annealing treatment). Note that the substrate peaks were not displayed for all samples because the grazing incident X-ray diffraction technique was used. The asterisk in the figure indicates the Mn_5_Si_3_ (ICSD card no. 04–003-4114) phase. For the MnSi film on Si (001), MnSi peaks were mainly observed; in addition, five peaks matched with the Mn_5_Si_3_ phase and several unknown impurity peaks were detected. However, we found that the peaks related to the Mn_5_Si_3_ phase were suppressed and the unknown peaks disappeared for MnSi on Al_2_O_3_ #1. Furthermore, the MnSi on Al_2_O_3_ #2 sample, in which the Mn power and annealing temperature decreased to 10 W and 550 °C, respectively, showed only MnSi (ICSD card no. 04–004-7568) peaks.

**Fig. 2:**
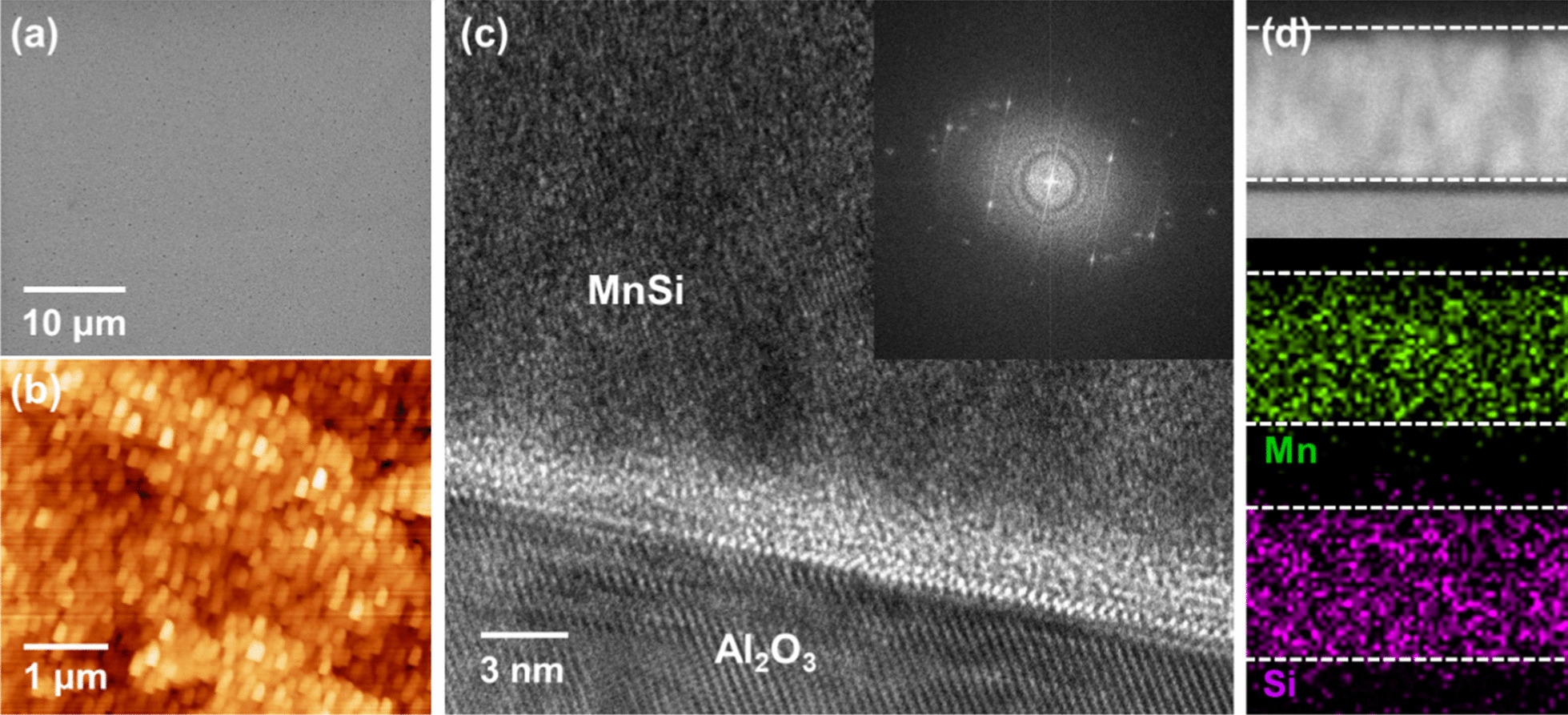
Morphological and structural characterization of MnSi film grown on Al_2_O_3_ substrate. **a** SEM image of the as-grown MnSi film. **b** AFM topographic image corresponding to **a**. RMS roughness is estimated to be under 1 nm. **c** Representative HR-TEM image of MnSi film grown on sapphire. Inset: FFT from selected area of MnSi in the HR-TEM image. **d** Elemental mapping of EDS of the cross-sectional MnSi film

Although the as-grown MnSi on Al_2_O_3_ #2 showed a somewhat defective surface, a highly uniform and low uneven surface was observed, as shown in the SEM image of Fig. 2a and the AFM topographic image of Fig. 2b. On the 15 × 15 μm scale of the AFM image, the root-mean-squared (RMS) roughness was measured to be under 1 nm. To characterize the detailed structure and chemical composition, cross-sectional TEM analyses of as-grown MnSi on Al_2_O_3_ #2 were carried out. Figure 2c shows a representative cross-sectional TEM image of MnSi on Al_2_O_3_ #2 at the interfacial region. Note that no stacking faults or significant defects were observed. When MnSi films are grown by conventional sputtering in a relatively low vacuum chamber, it is hard to expect that MnSi grows epitaxially to the preferred direction of the surface of substrates, considering structural parameters such as lattice mismatch and chemical bonding. Our MnSi films grown on Al_2_O_3_ have a polycrystalline nature, as confirmed by XRD patterns (Fig. 1) and fast Fourier transform (FFT) of the TEM image [inset of Fig. 2c]. We examined the chemical composition of the as-grown MnSi films. As seen in the TEM-EDS mapping of Fig. 2d, the presence of only Mn and Si elements was detected in several different regions, and the atomic ratio of Mn/Si = 1:1.1 was estimated. We tested the growth rate of MnSi films by controlling the growth time. The thickness of the as-grown MnSi films showed a linear behavior for the growth time (Additional file 1: Fig. S2).

**Fig. 3:**
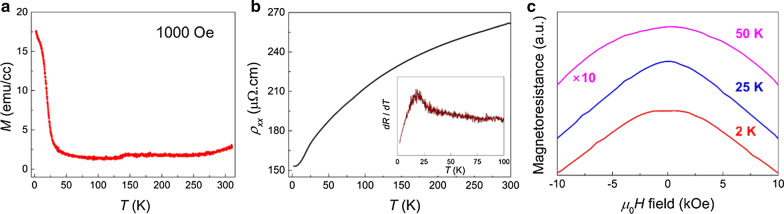
**a** Field-cooled magnetization as a function of temperature for a 25 nm thick MnSi film in an external magnetic field of 1 kOe. **b** Zero-field longitudinal resistance as a function of temperature. Inset: derivative of the resistance as a function of the temperature highlighting the anomaly of magnetic transition. **c** Perpendicular magnetoresistance at 2, 25, and 50 K. For clarity, arbitrary offsets are added, and the magnetoresistance measured at 50 K is magnified by 10 times

Figure 3a shows the temperature dependence of magnetization for MnSi on Al_2_O_3_ (thickness 25 nm) measured in an out-of-plane magnetic field of 1 kOe. The magnetization dropped significantly at temperatures above 25 K, indicating a ferromagnetic transition temperature (*T*_C_), similar to bulk MnSi [26, 27]. The resistivity depending on the temperature exhibited metallic behavior above *T*_C_, as shown in Fig. 3b. Below *T*_C_, the resistivity tended to decrease with *T*^2^ dependence as the temperature decreased, owing to the coupling of charge carriers to spin fluctuations in the helimagnetic phase [28]. As seen in the inset of Fig. 3b, the derivative of resistivity versus temperature highlighted the *T*_C_ of MnSi films at approximately 25 K. The polycrystals and defects on the surface give rise to a low residual resistivity ratio, i.e., [*ρ*(300 K)/*ρ*(5 K)] ~ 1.7.

Figure 3c shows the magnetoresistance for the magnetic fields perpendicular to the film plane at different temperatures of 2 K, 25 K, and 50 K. As we discussed above, since the as-grown MnSi films had a polycrystalline nature, the magnetic phase transition from the magnetoresistance was not clearly observed. In low magnetic fields, however, the temperature dependence of the magnetoresistance exhibited distinguishable features. As the temperature increased, the shape of the magnetoresistance in the vicinity of the zero magnetic field changed from flat (2 K) to sharp (25 K) and broad (50 K) peaks.

Regarding the spin-chirality-driven Hall effect, THE can be induced by DMI arising from strong spin–orbit coupling and non-centrosymmetric B20 crystal structure [29], which is considered a hallmark of the existence of the skyrmion phase. We performed Hall resistivity measurements to observe abnormal resistivity related to THE. The total Hall resistivity can be expressed as a combination of three components:

$$\begin{aligned} \rho_{{{\text{Hall}}}} & = \rho_{{{\text{normal}}}} + \rho_{{{\text{AHE}}}} + \rho_{{{\text{THE}}}} \\ & = R_{0} H + \left( {\alpha \rho_{xx0} + \beta \rho_{xx0}^{2} + b\rho_{xx}^{2} } \right)M + n_{{{\text{Skx}}}} PR_{{{\text{TH}}}} B_{{{\text{eff}}}} , \\ \end{aligned}$$

where *ρ*_normal_, *ρ*_AHE_, and *ρ*_THE_ are the normal, anomalous, and topological Hall resistivities, respectively. *R*_0_ is the normal Hall coefficient, and *α*, *β*, and *b* are the constants corresponding to the skew scattering, side jump, and intrinsic contributions to the anomalous Hall resistivity. Additionally, *n*_Skx_ is the relative skyrmion density, *P* is the polarization of the conduction electrons, *R*_TH_ is the topological Hall coefficient, and *B*_eff_ is the effective magnetic field derived from the real-space Berry phase [20, 30]. The topological Hall contribution can be extracted by subtracting the normal and anomalous Hall resistivity terms from the measured total Hall resistivity.

**Fig. 4:**
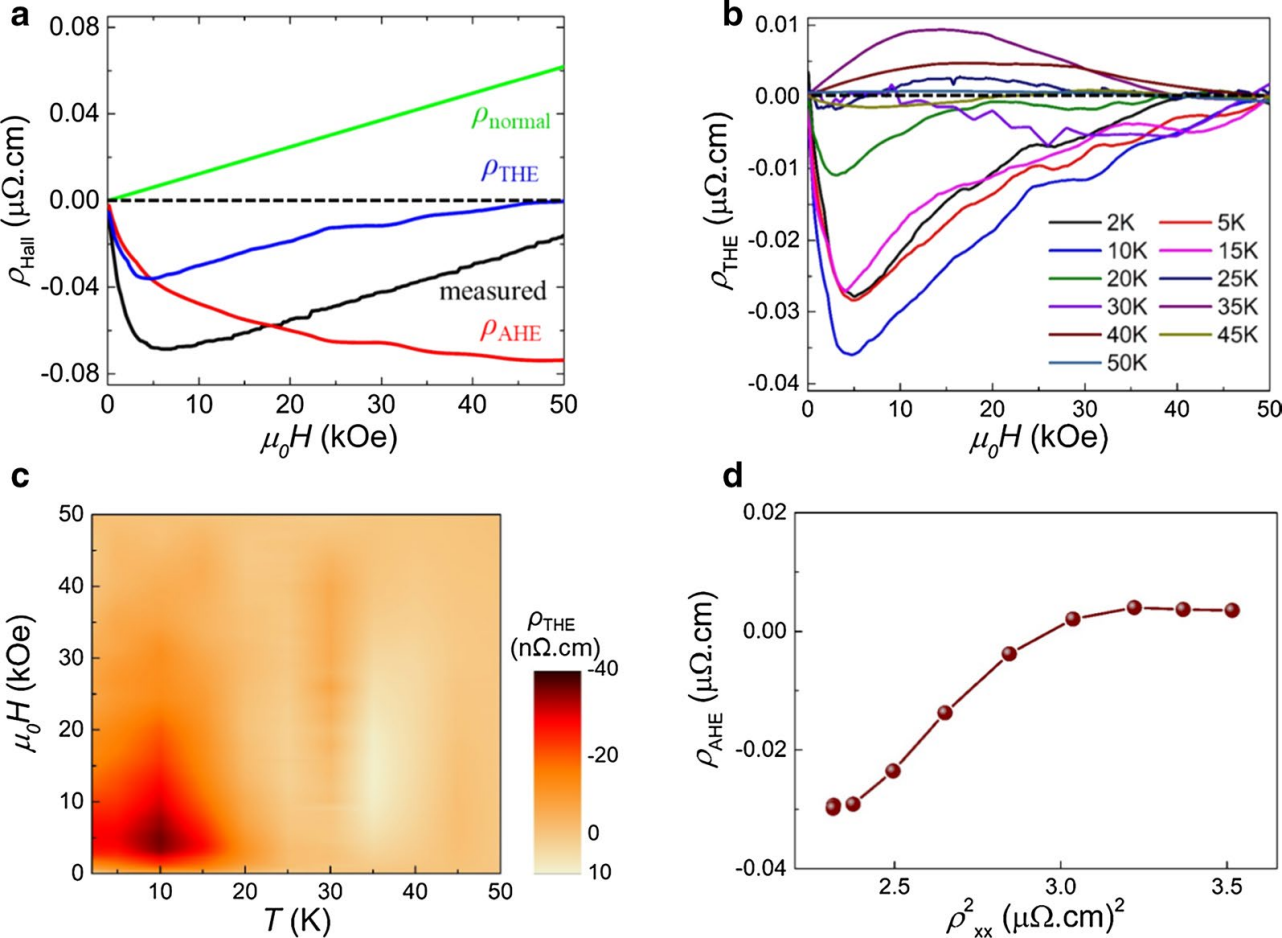
**a** The representative Hall resistivity curve at 10 K. The THE signal (blue curve) is extracted by the subtracting normal (green line) and anomalous Hall signals (red curve) from the total measured Hall resistivity (black curve). **b** Topological Hall resistivities at various temperatures, extracted using the same procedure detailed in the text. **c** The contour mapping of the THE signal as a function of the magnetic field and temperature, constructed by interpolation of topological Hall resistivity between temperatures. **d** Anomalous Hall resistivity as a function of the squared longitudinal magnetoresistivity below the temperature where the topological Hall resistivity is not zero

Figure 4a shows deconvoluted Hall data to extract the THE signal at 10 K as the blue curve, including normal (green line) and anomalous (red curve) Hall resistivities. Note that the positive slope of *ρ*_normal_ indicates *p*-type majority carriers, and *ρ*_AHE_ is negative, consistent with those of bulk MnSi [31], thin films [9], and nanowires [20]. *ρ*_normal_ is obtained from the linear fit at high magnetic fields, and *ρ*_AHE_ is directly taken from the magnetization data. The *ρ*_THE_ depending on the temperature is displayed in Fig. 4b. Interestingly, the sign of *ρ*_THE_ flipped at the border of 25 K, where the magnetic transition was expected. The sign of *ρ*_THE_ is very sensitive to the spin polarization of charge carriers. In the band structure of MnSi, the localized electrons in the *d* band affect the density of states near the Fermi level, while itinerant electrons in the *s* band contribute meagerly to the band structure [31], allowing the spin polarization to be delicate. In addition, since the spin polarization can be changed by external factors such as tensile strain and crystal purity with temperature [9], the flipped sign of *ρ*_THE_ in our polycrystalline MnSi sample is reasonable. Figure 4c presents the contour mapping of *ρ*_THE_ as a function of magnetic field and temperature. While the skyrmion phase in bulk MnSi was observed in a narrow temperature range close to the magnetic transition temperature, a nonzero *ρ*_THE_ was collected from 2 to 40 K regardless of the sign. The absolute value of *ρ*_THE_ had a maximum of 36 nΩ cm at 10 K and 4 kOe, larger than that of thin films grown by MBE (10 nΩ cm) [9], bulk (4.5 nΩ cm) [32], and nanowire (15 nΩ cm) [20] but similar to that of thin films grown by off-axis magnetron sputtering with an ultrahigh vacuum chamber [25].

*ρ*_AHE_ consists of three components: skew scattering, side jump, and intrinsic contributions. An implication in the scaling of the anomalous Hall contribution is that *ρ*_AHE_ is proportional to the intrinsic contribution, $\rho_{xx}^{2}$, associated with the momentum-space Berry phase [33]. In Fig. 4d, we plot *ρ*_AHE_ against $\rho_{xx}^{2}$ at 20 kOe, showing an obvious deviation from linear dependence. The breakdown of the scaling suggests that the anomalous Hall effect is relevant to extrinsic skew scattering and side jump contributions caused by impurities and defects in our polycrystalline MnSi sample, retaining the stabilization of the skyrmion phase in a broader range of temperatures and magnetic fields.

## Conclusion

In summary, we demonstrated a method to grow MnSi films on Al_2_O_3_ by conventional magnetron sputtering with a relatively low vacuum chamber. Developing a facile way to fabricate various nanostructures is imperative [34, 35]. The spectroscopic and morphological analyses confirmed that the as-deposited MnSi films have a polycrystalline nature with a highly uniform and low roughness surface. The transport properties exhibit the intrinsic characteristics of MnSi, although the magnetic transition temperature was slightly lower than that of previous results. More importantly, we observe a stable skyrmion phase in a broad range of temperatures and magnetic fields, even in our polycrystalline MnSi films, attributed to the complicated implication of the Hall resistivity contribution. This work opens up the opportunity for extensive investigation of materials possessing skyrmion phases beyond the burden of preparing single crystals or epitaxial thin films.

## Supplementary Information

**Additional file 1:** Supplementary information.

## Data Availability

All data generated or analyzed during this study are included in this published article and its supplementary information files, and are available from corresponding author on reasonable request.

## Abbreviations

DMI: Dzyaloshinskii–Moriya interaction
STT: Spin transfer torque
THE: Topological Hall effect
XRD: X-ray diffraction
TEM: Transmission electron microscope
Al_2_O_3_: Sapphire
DC: Direct current
RF: Radio frequency
SEM: Scanning electron microscopy
AFM: Atomic force microscopy
HR-TEM: High-resolution transmission electron microscopy
EDS: Energy-dispersive spectroscopy
SQUID-VSM: Superconducting quantum interference device-vibrating sample magnetometer
RMS: Root-mean-squared
FFT: Fast Fourier transform
*T*_C_: Ferromagnetic transition temperature
