# Supplementary material for: Skyrmion Phase in MnSi Thin Films Grown on Sapphire by a Conventional Sputtering
Source: Nanoscale Res Lett. 2021 Jan 6;16:7. doi: 10.1186/s11671-020-03462-2 (PMC7788108; doi:10.1186/s11671-020-03462-2)
Supplement: Supplementary file 1 — Additional file 1: Supplementary information. [file 11671_2020_3462_MOESM1_ESM.docx]

**Supplementary information**

**Skyrmion phase in MnSi thin films grown on sapphire by a conventional sputtering**

Won-Young Choi ^a^, Hyun-Woo Bang ^a^, Seung-Hyun Chun ^b^, Sunghun Lee ^b,*^, and Myung-Hwa Jung ^a,*^

^a^ Department of Physics, Sogang University, Seoul 04107, Korea

^b^ Department of Physics, Sejong University, Seoul 05006, Korea

^*^E-mail: [kshlee@sejong.ac.kr](mailto:kshlee@sejong.ac.kr)(S.L.) or [mhjung@sogang.ac.kr](mailto:mhjung@sogang.ac.kr)(M.H.J.)

**KEYWORDS:** MnSi, Sputtering, Polycrystal, Skyrmion, Topological Hall effect

**
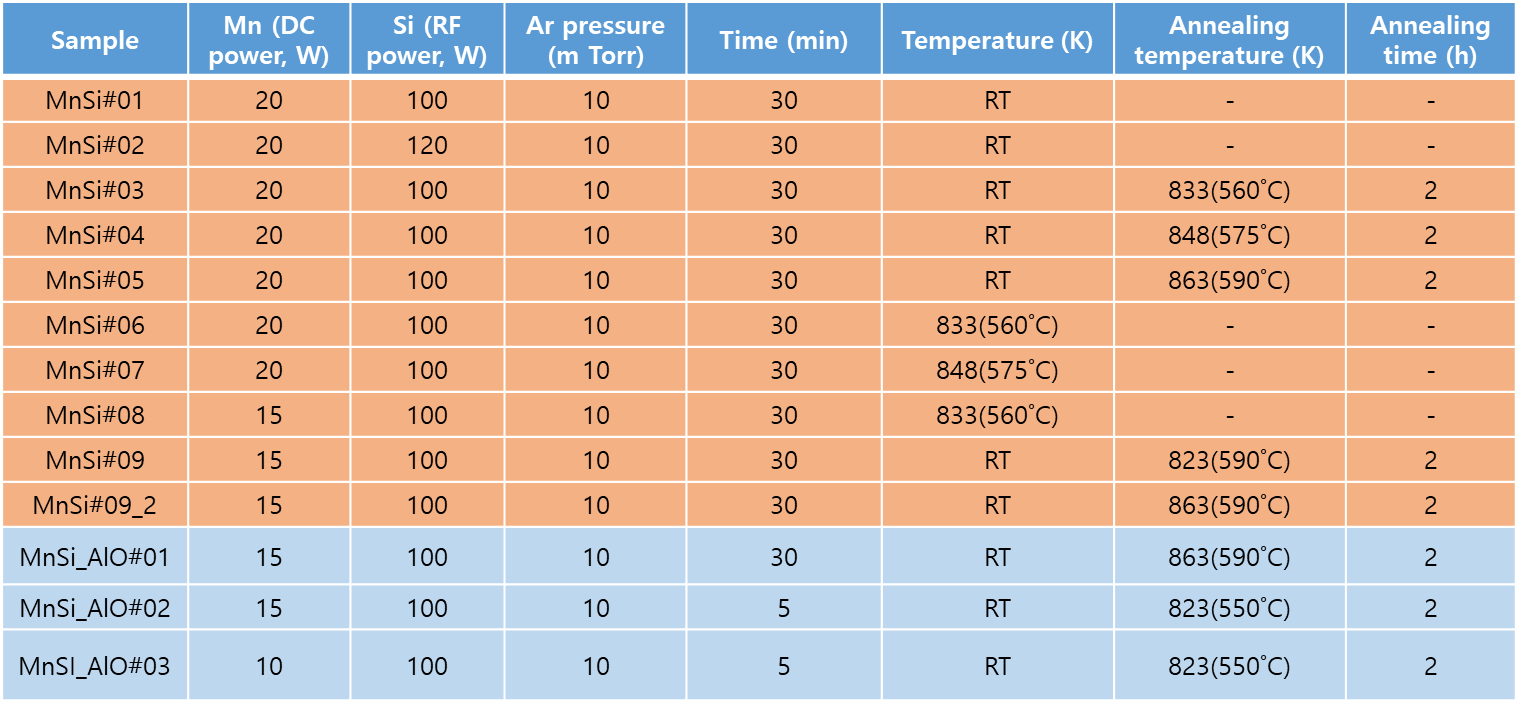
**

**Table S1.** Growth conditions for MnSi on Si (001) and Al_2_O_3_ substrates, varying Mn power/growth temperature/annealing temperature.


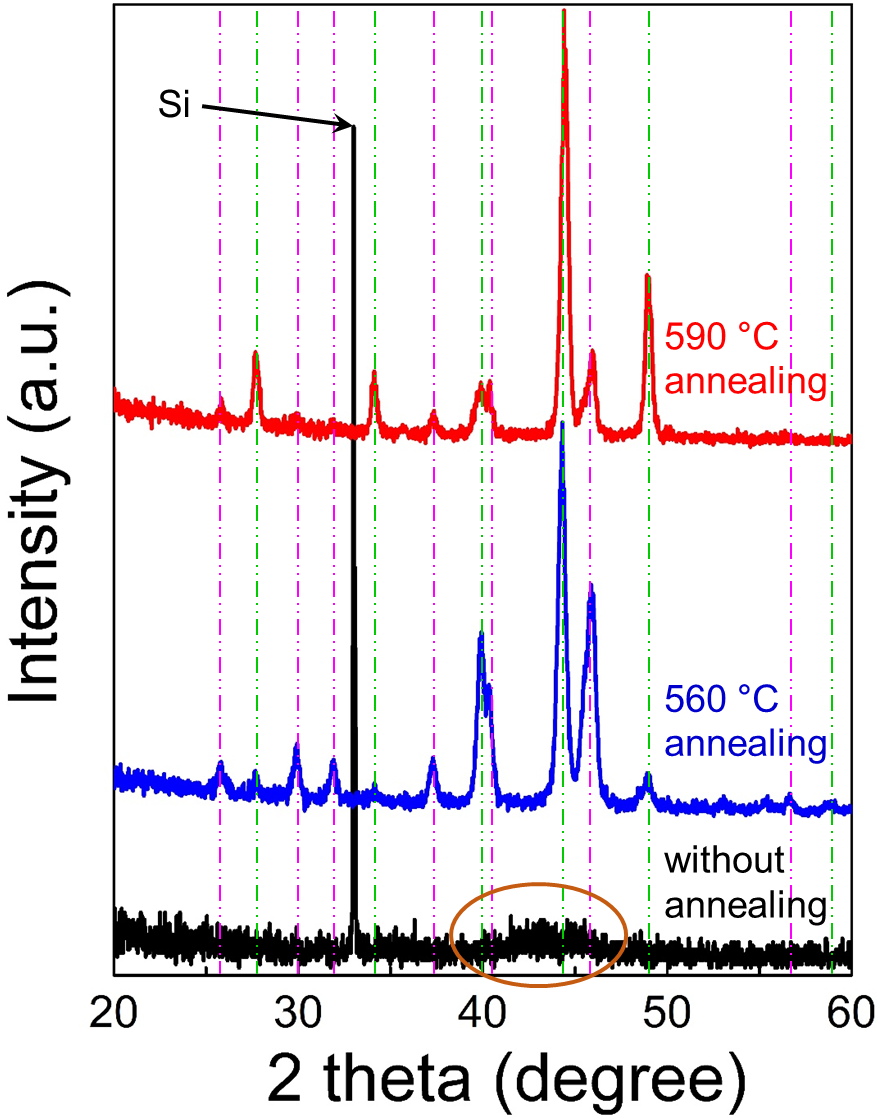


**Figure S1.** XRD patterns of MnSi films on Si (001) substrate. By examining the annealing treatment, initially deposited amorphous MnSi (brown open circle in black XRD line) turned into crystallized MnSi phase after annealing treatment over specific temperature. Magenta and green dotted lines indicate Mn_5_Si_3_ and MnSi phase, respectively.

**
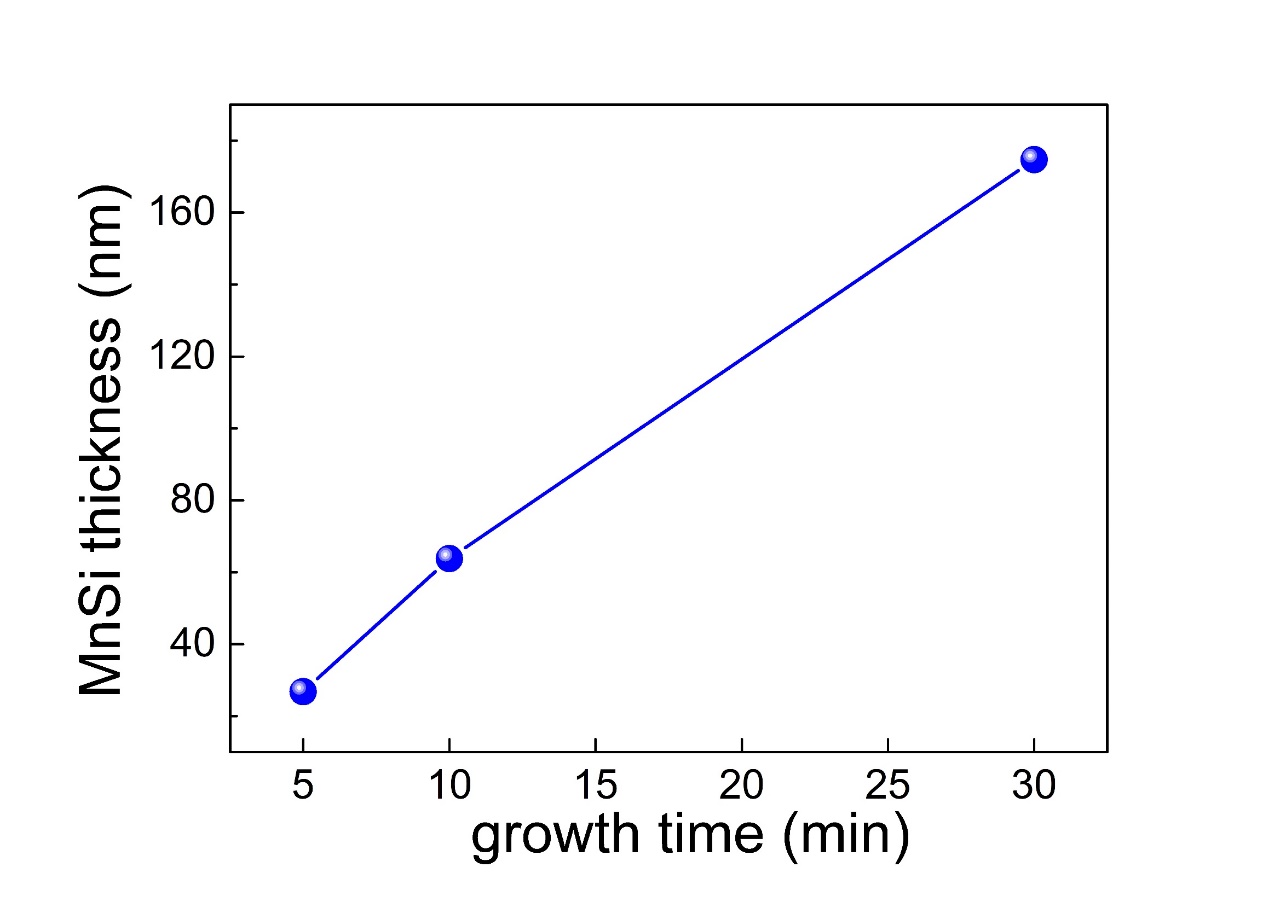
**

**Figure S2.** Thickness of MnSi films regarding of growth time in conventional sputtering, showing linear behavior.
